# Flagellin synergistically enhances anti-tumor effect of EGFRvIII peptide in a glioblastoma-bearing mouse brain tumor model

**DOI:** 10.1186/s12885-022-10023-6

**Published:** 2022-09-15

**Authors:** Jin Myung Choi, Sa-Hoe Lim, Zhi-Peng Liu, Tae Kyu Lee, Joon Haeng Rhee, Mee Sun Yoon, Jung-Joon Min, Shin Jung

**Affiliations:** 1 Brain Tumor Research Laboratory, Biomedical Research Institute, Chonnam National University Hwasun Hospital, Hwasun, 58128 Republic of Korea; 2 Department of Neurosurgery, Chonnam National University Hwasun Hospital &School of Medicine, 322 Seoyang-ro, Hwasun, 58128 Republic of Korea; 3 Department of Microbiology, Chonnam National University Medical School, Hwasun, 58128 Republic of Korea; 4 Department of Radiation Oncology, Chonnam National University Hwasun Hospital &Medical School, Hwasun, 58128 Republic of Korea; 5 Department of Nuclear Medicine, Chonnam National University Medical School, Hwasun, 58128 Republic of Korea

**Keywords:** Glioblastoma, Immunotherapy, EGFRvIII, Flagellin, Adjuvant

## Abstract

**Background:**

Glioblastoma (GBM) is the most aggressive type of brain tumor with heterogeneity and strong invasive ability. Treatment of GBM has not improved significantly despite the progress of immunotherapy and classical therapy. Epidermal growth factor receptor variant III (EGFRvIII), one of GBM-associated mutants, is regarded as an ideal therapeutic target in EGFRvIII-expressed GBM patients because it is a tumor-specific receptor expressed only in tumors. Flagellin B (FlaB) originated from *Vibrio vulnificus*, is known as a strong adjuvant that enhances innate and adaptive immunity in various vaccine models. This study investigated whether FlaB synergistically could enhance the anti-tumor effect of EGFRvIII peptide (P_EGFRvIII_).

**Methods:**

EGFRvIII-GL261/Fluc cells were used for glioblastoma-bearing mouse brain model. Cell-bearing mice were inoculated with PBS, FlaB alone, P_EGFRvIII_ alone, and P_EGFRvIII_ plus FlaB. Tumor growth based on MRI and the survival rate was investigated. T cell population was examined by flow cytometry analysis. Both cleaved caspase-3 and CD8 + lymphocytes were shown by immunohistochemistry (IHC) staining.

**Results:**

The P_EGFRvIII_ plus FlaB group showed delayed tumor growth and increased survival rate when compared to other treatment groups. As evidence of apoptosis, cleaved caspase-3 expression and DNA disruption were more increased in the P_EGFRvIII_ plus FlaB group than in other groups. In addition, the P_EGFRvIII_ plus FlaB group showed more increased CD8 + T cells and decreased Treg cells than other treatment groups in the brain.

**Conclusions:**

FlaB can enhance the anti-tumor effect of P_EGFRvIII_ by increasing CD8 + T cell response in a mouse brain GBM model.

**Supplementary Information:**

The online version contains supplementary material available at 10.1186/s12885-022-10023-6.

## Background

Glioblastoma (GBM), the most common primary malignant brain tumor in adults, has a low overall survival (OS, < 15 months) after its diagnosis [1]. Up to now, treatments for patients with GBM mainly depend on common classical methods such as surgery, radiation, and chemotherapy without leading to much improvement [2]. The microenvironment of GBM has characteristics of a cold tumor that was formed in the absence of T cell infiltration and the induction of immune suppressive cells such as regulatory T cell, M2 type macrophage, and myeloid-derived suppressor cells (MDSC) in tumor. Cytokines secreted from immune-suppressive cells such as transforming growth factor-β(TGF-β and interleukine-10 (IL-10) can inhibit effector T cells and promote tumor growth [3]. Thus, many research groups have recognized the necessity of immunotherapy for GBM. The purpose of using immunotherapy for GBM treatment is to induce tumor suppression by changing the tumor microenvironment from ‘cold’ to ‘hot’ through increasing cytotoxic T cells, M1 type macrophages, and APC while reducing Treg. To induce a change in the microenvironment, many researchers are developing various immunotherapies such as a tumor-specific peptide, dendritic cell (DC) vaccine, chimeric antigen receptor (CAR)-T cell, and checkpoint inhibitors [4].

Epidermal growth factor receptor variant III (EGFRvIII), one of the GBM-associated mutations, is a variant of EGFR with deletion of amino acids 6–273, leading to the deletion of EGFR extracellular domain (exon 2 to 7) [5]. It is a tumor-specific receptor that is only expressed on the surface of tumor cells and not on normal cells. Approximately 30% of patients with GBM express the EGFRvIII gene [6]. Thus, EGFRvIII is an ideal target of immunotherapy in EGFRvIII-expressing GBM patients as well as breast, ovarian, and glial tumors [7]. Until now, several peptide-based immunotherapies targeting EGFRvIII have been subjected to both preclinical and clinical trials. In preclinical trials, Heimberger et al. have reported that PEP-KLH vaccination can generate potent anti-tumor effects against subcutaneous and intracerebral tumors [8]. Wu et al. [9] have demonstrated that EGFRvIII-derived cytotoxic T lymphocyte (CTL) epitopes restricted by HLA A0201 (MHC class I) can induce a cytotoxic immune response in gliomas. In clinical trials, rindopepimut (CDX-110) has been administrated to newly diagnosed GB patients. The safety and efficacy of rindopepimut in Phase I and Phase II trials have been confirmed [10, 11]. In phase 1 clinical trial, PFS and OS of patients with rindopepimut after resection and radiotherapy was 6.8 months and 18.7 months. In phase 2 clinical trial, PFS and OS of patients with rindopepimut after radiotherapy and concurrent TMZ was 14.2 months and 26 months vaccination [12].

Flagellin, an agonist of toll-like receptor 5 (TLR5), is known as a potential adjuvant that can induce innate and adaptive immune responses of host cells [13–15]. TLR5 is expressed on the surface of immune-related cells including monocytes, macrophages, neutrophils, lymphocytes, NK cells, and dendritic cells (DCs) [16, 17]. Flagellin administrated by IN injection showed colocalization with putative dendritic cells and increased TLR5-expressing cells in cervical lymph nodes [18]. Cai et al. [19] have reported that flagellin can suppress cell proliferation and tumor growth by activating TLR5 on breast cancer cells. Rhee et al. [20] have demonstrated that flagellin could inhibit tumor growth through its strong antitumor activity in colon cancer. Nguyen et al. [21] have also reported that flagellin can enhance tumor antigen (TA)—specific CD8 + T cell immune response in a therapeutic cancer vaccine model. Dong et al. [22] have described that intracranial injection of flagellin plus tumor cell lysate (TCL) can enhance survival by recruiting CD4 + and CD8 + T cells to brain tissues in a GL261-bearing C57BL/6 mice GBM model.

In this study, we investigated whether Flagellin B (FlaB) could enhance the anti-tumor immunity in a GL261 glioma mouse brain model when it was combined with peptide-based immunotherapy. EGFRvIII peptide (P_EGFRvIII_) used in our experiment is consisted of nine amino acids (LEEKKGNYV) and is known as the best epitope for MHC I binding to induce tumor-specific immune response [9]. Intranasal (IN) administration by inhalation more effectively delivered P_EGFRvIII_ and FlaB to the brain than intravenous (IV) or intraperitoneal (IP) injection. P_EGFRvIII_ plus FlaB group inhibited tumor growth and prolonged survival by changing CD8 + /Treg ratio compared to single treatment groups. Here, we propose that flagellin can enhance the anti-tumor effect of a peptide through CD8 + T cell-mediated immune response in a mouse brain GBM model.

## Materials and methods

### Cell line, Flagellin B, and EGFRvIII peptide

Murine GL261 glioma cells transfected with firefly luciferase (GL261/Fluc) were obtained from Dr. Kang (Yonsei-University, South Korea). The human EGFRvIII gene was purchased from a company (Addgene, USA). Human EGFRvIII-overexpressing murine GL261/Fluc glioma cells (EGFRvIII-GL261/Fluc) were manufactured by transfection using a lentivirus vector system obtained from Dr. Min (Chonnam University Hwasun Hospital, South Korea). Cells were cultured in high-glucose Dulbecco’s Modified Eagle’s Medium (DMEM) supplemented with 10% fetal bovine serum and 1% penicillin/streptomycin in a humidified atmosphere with 5% CO_2_ at 37 °C. Recombinant Flagellin B (FlaB) was provided by Dr. Rhee (Chonnam National University medical school, South Korea). Synthetic P_EGFRvIII_ (9 mer, LEEKKGNYV) was purchased from a company (Anygen, South Korea).

### Mouse intracranial tumor model and treatment condition

EGFRvIII-GL261/Fluc cells were used for preparing a mouse brain tumor model. Seven-week-old female C57BL/6 mice purchased from a company (Orient Bio, South Korea) were anesthetized with a mixture of zoletil and rompun via intramuscular (i.m) injection. A burr hole was drilled on the surface at 2 mm posterior and 2 mm right from the bregma. Then 2 × 10^5^ of cells (2 ul) were stereotactically injected into a depth 3 mm of the right hemisphere with a sterile 10 ul Hamilton syringe. After injection into the brain, the small hole was sealed with bone wax and sutured by the scalp. Mice were intranasally immunized three times by inhalation of nose (each side 10ul) with 5 ug FlaB alone, 15 ug P_EGFRvIII_ alone, or 5 ug FlaB plus 15 ug P_EGFRvIII_ in 20ul of phosphate-buffered saline (PBS) under anesthesia at 5-day intervals. The detailed treatment schedule is presented in Figs. 3a and 4a.

### Western blotting

EGFRvIII-GL261/Fluc cells were treated with and without PI3K inhibitor (LY294002), and then cultured for 6 h. The harvested cells were lysed by RIPA buffer containing Tris–HCl, pH 7.5, 1% Triton X-100, 0.1 M NaCl, 0.5% sodium deoxylcholate, 2 mM EDTA (Biosolution). Proteins extracted from whole cell lysates(30ug) were run by using electrophoresis with 10% SDS–polyacrylamide gel at 100 V for 2 h, and then transferred to PVDF membranes at 100 V for 1 h on ice. The membranes were blocked for 1 h at room temperature with 5% non-fat dry milk, incubated at 4 °C for overnight with following primary antibodies; β-actin (1:2000, CST), AKT (1:1000, CST), p-AKT(Ser473) (1:1000, CST), PI3K (1:1000, CST), and p-PI3K(Tyr458) (1:1000, CST) rabbit monoclonal antibodies. Secondary antibodies were a horseradish-labled goat anti-rabbit IgG (1:3000, CST). Protein was detected by using Amersham 6000 (Amersham Biosciences).

### Tumor formationand delivery of syntheticCe6-P_EGFRvIII_ and FlaB

Tumor images of mice were obtained using an animal magnetic resonance imaging (MRI) machine (M7™ compact MRI scanner). Enhancer (Magnevist, BAYER, Germany) was injected into the tail vein for tumor identification with an anesthetic. Tumor volume was calculated using Oxrix Lite ver.12.0.1 software. P_EGFRvIII_ conjugated with chlorin e6 (Ce6) was prepared to investigate the delivery of peptide into the brain. For the preparation of Ce6- P_EGFRvIII_, 6 mg Ce6, 1.92 mg EDTA, and 1.15 mg N-hydroxysuccinimide (NHS) were mixed in 1 ml dimethyl sulfoxide (DMSO). This solution was incubated at room temperature (RT) for 6 h reaction by stirring in a dark condition and then mixed with 10 mg peptide in 2 ml PBS. The mixed solution was incubated at RT for 12 h reaction by stirring in a dark condition. The mixture was transferred into a dialysis membrane and dialyzed in 1 L DW for 1 day. After dialysis, Ce6-P_EGFRvIII_ was used for administration. A mixture of Ce6-P_EGFRvIII_ and FlaB (total 20 ul) was injected into the intranasal channel for delivering P_EGFRvIII_ and FlaB to the brain. To identify the movement of P_EGFRvIII_ and FlaB, the brains of sacrificed mice was extracted at 4 h after injecting the mixture. Fluorescence signals of Ce6-P_EGFRvIII_ were detected with an in vivo imaging system (IVIS) spectrum (PerkinElmer). Pseudo-color images indicating photon counts were analyzed using Living Image software v. 4.7.2 (PerkinElmer).

### Immunofluorescence and immunohistochemistry staining

Extracted mice brains were fixed in 4% formaldehyde solution. Hematoxylin and Eosin (H&E) stained and paraffin-embedded slides were prepared by the department of pathology, Chonnam University Hwasun Hospital. For immunofluorescence (IF) staining of FlaB, paraffin-embedded specimens were dewaxed in xylene for 15 min, hydrated with ethanol (100, 80, 60%) for 10 min each, and then washed with distilled water for 10 min. Specimens were soaked in EnVision FLEX target retrieval low pH solution at heated with high pressure for 15 min to perform heat-mediated antigen retrieval. After natural cooling, endogenous peroxidase activity was quenched with a peroxidase-blocking solution (Dako) for 30 min. After PBST (1xPBS + 0.5% Tween20) washing for 5 min three times, nonspecific sites were blocked with antibody diluent solution (Dako) for 30 min. Mouse anti-FlaB (1:200) was used as the primary antibody for FlaB detection. Anti-mouse Alexa Fluor 488 (Green, 1:200, Invitrogen) for FlaB was used as the secondary antibody. 4’,6-diamidino-2-phenylindole (DAPI) staining was performed as counterstaining. Samples were mounted with 4′, 6-diamidino-2-phenylindole/Antifade. Images of fluorescent immunolabeled sections were obtained using a fluorescence microscope. For immunohistochemistry (IHC) staining, slides were proceeded with a bond primary refine detection kit (Leica Biosystems) following the manufacturer’s protocol using Leica Bond. Primary antibodies against cleaved caspase-3 (CC-3, 1:1000; Cell Signaling Technology) and CD8a (1:400; Cell Signaling Technology) were used.

### Immune cell isolation from tissues and flow cytometry

To isolate single immune cells from a mouse brain tumor and lymph node, organs were extracted and were mixed with fetal bovine serum (FBS)-free RPMI 1640. Mashed tissues by syringe rubber were passed through a 100-um nylon cell strainer with FBS-free RPMI 1640. After centrifugation at 1400 rpm for 5 min at 4 ℃, the supernatant was discarded. The brain pellet was incubated with 1 ug/ml DNAase I and 0.05% collagenase I at 37℃ for 45 min with shaking (200 rpm). Aggregated debris was removed by passing through a 70-um nylon cell strainer. After centrifugation at 1400 rpm for 5 min at 4 °C, the pellet was resuspended in 5 mL of 30% Percoll (GE) and overlaid on the top of a gradient containing 5 mL of 75% Percoll solution. The gradient was centrifuged at 2000 rpm for 20 min at RT. Cells were collected from the middle interface of Percoll solution and washed once with fluorescence-activated cell sorting (FACS) buffer. The procedure for single-cell isolation from lymph node was the same except for enzyme and Percoll gradient. After cell counting, cells were transferred into a 96-well microplate. For surface staining, cells were incubated with the following antibodies at 4 °C for 30 min: live/dead (1:1000; BD), Pacific Blue-CD3(1:300; BD), PE-CD4 (1:300; BD), APC-CD8 (1:300; BD). For intranuclear staining, cells were incubated at 4 °C with permeabilization solution (Fixation 1: permeabilization 3, eBioscience) for 60 min. After centrifugation at 4 °C and 1400 rpm, the supernatant was discarded and cells were incubated with diluted PE-Foxp3 (1:300; BD eBioscience) at 4 °C for 20 min.

### Statistical analysis

All statistical analyses were performed using GraphPad Prism 6.0 software. Statistical significance was considered at *p* value < 0.05. Survival analysis was performed using the Kaplan–Meier method and the log-rank test. All data are expressed as means ± standard error of the mean (SEM).

## Results

### Characterization of EGFRvIII-GL261/Fluc cells

**Fig. 1:**
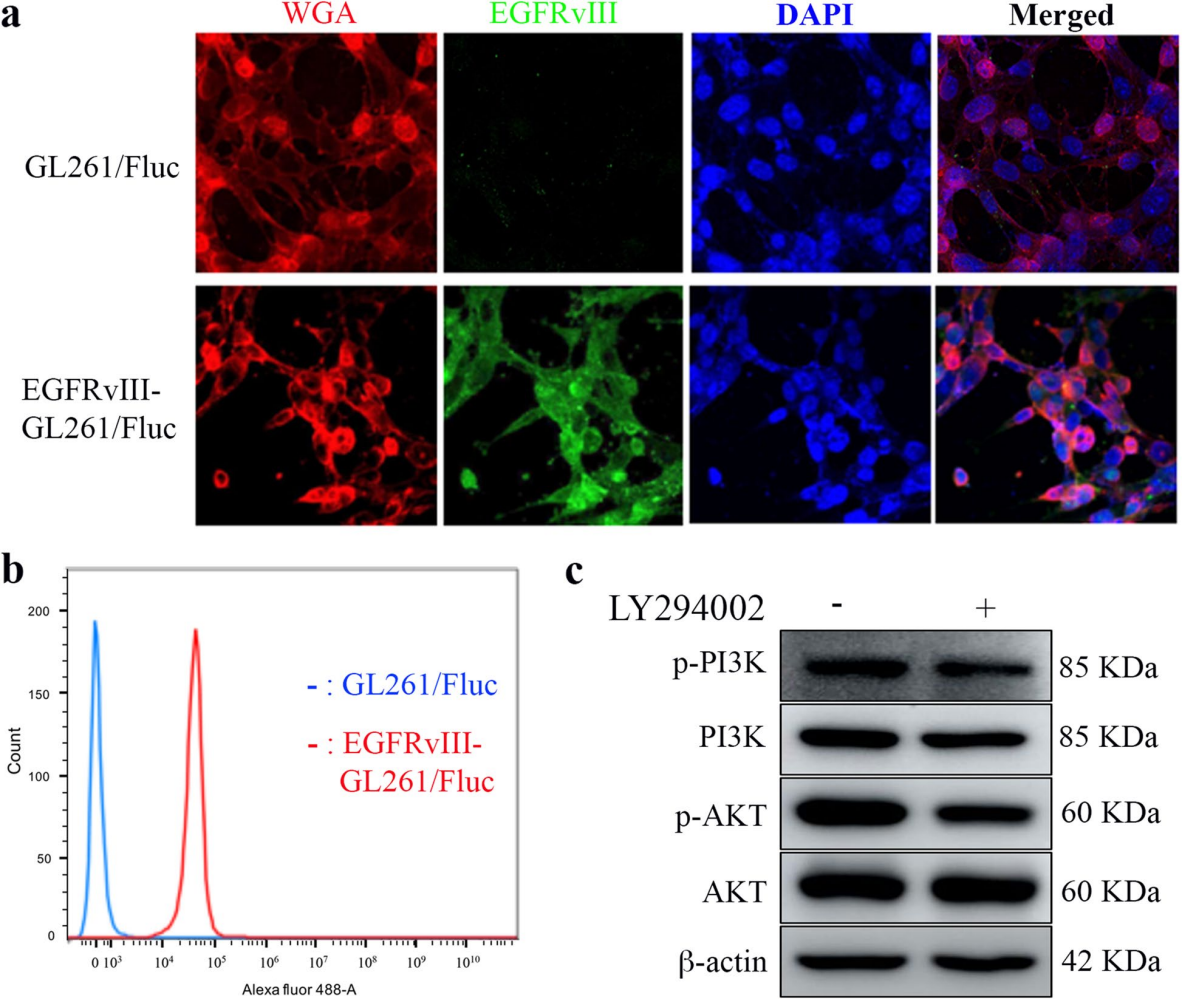
Characterization of EGFRvIII-GL261/Fluc cells. **a** Both GL261/Fluc and EGFRvIII-GL261 cells were double-stained with WGA (wheat Germ Agglutinin, red) and EFGRvIII (green). Nuclei were stained with DAPI (blue).** b** EGFRvIII expression on the cell surface was detected with Alex488 by flow cytometry.** c** EGFRvIII- GL261/Fluc cells were treated without (-) or with ( +) LY294002 (PI3K inhibitor) to identify the kinetic activity of EGFRvIII. Phosphorylation levels of PI3K and AKT from cell lysates were analyzed by western blotting using p-PI3K p85(Y458) and pAKT(S473) antibodies. Full-length blots/gel are presented in Supplementary Fig. 1

The human EGFRvIII gene was transfected into GL261/Fluc cells using a lentiviral vector system to generate EGFRvIII-GL261/Fluc cells. We first examined whether EGFRvIII was expressed on EGFRvIII-GL261/Fluc cells. As shown in Fig. 1, we confirmed the position of EGFRvIII on the cell surface through the co-localization of WGA staining with EGFRvIII by IF staining and flow cytometry. In addition, cells were treated without or with LY294002 (PI3K inhibitor) to check whether EGFRvIII properly activated its downstream signals such as PI3K and AKT. After LY294002 treatment, phosphorylation levels of PI3K and AKT were decreased compared to without LY294002 (Fig. 1c, supple Fig. 1b). These results indicate that EGFRvIII was expressed on the cell surface and its downstream signals were functionally operated.

### Delivery of P_EGFRvIII_ and FlaBto mousebrain tumor

**Fig. 2:**
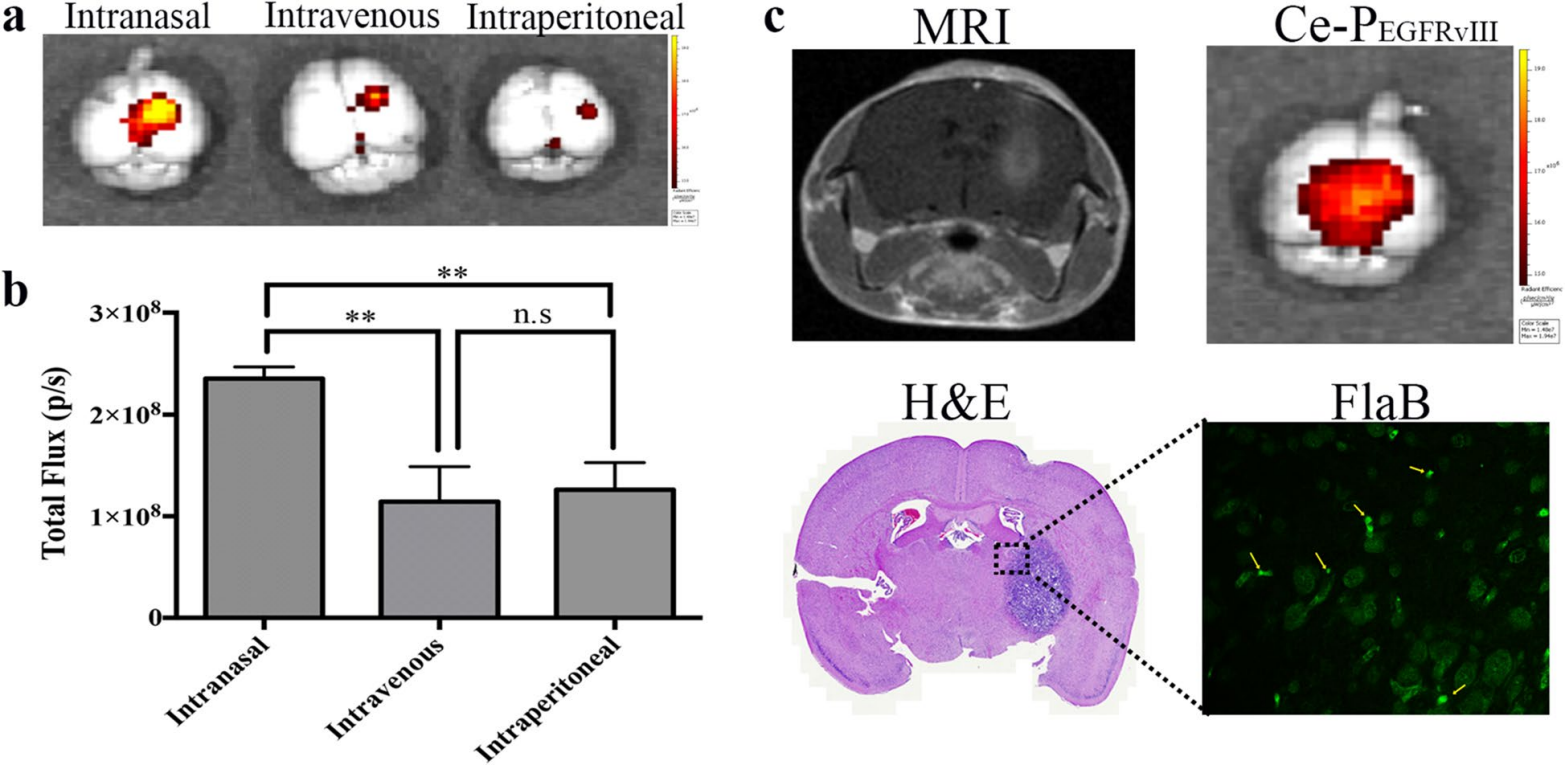
Delivery of P_EGFRvIII_ and FlaB to mouse brain tumor. **a** Ce6-P_EGFRvIII_ was administrated through different injection methods (intranasal; IN, intravenous; IV, intraperitoneal; IP) and the brain was extracted at 4 h after administration. Image of fluorescence of Ce6-P_EGFRvIII_ in brain tumor was obtained using IVIS spectrum at a wavelength between 640 and 700 nm. **b** Results are statistically analyzed and an average value of total flux (p/s) is shown (*n* = 3 for each group). **, *p* < 0.001. **c** Tumor is identified by MRI and H&E staining at 2 weeks after EGFRvIII- GL261/Fluc cell implantation. Synthetic Ce6-P_EGFRvIII_ and FlaB were mixed and IN injected (total 20 ul) to C57BL/6 mouse. Signals of Ce6-P_EGFRvIII_ in brains were detected by the IVIS spectrum. To identify FlaB delivery into the brain, paraffin-embedded tissue slides were prepared. FlaB was detected by IF staining using Alexa 488 (green). Each image represents one of three mice

Ce6-P_EGFRvIII_ was administered by three injection methods (intranasal or IN, intravenous or IV, and intraperitoneal or IP) to optimize the delivery condition of the peptide to the mouse brain tumor model. After extraction of the mouse brain, signals of Ce6-P_EGFRvIII_ on the brain were investigated at a wavelength between an exciting wavelength of 640 nm and an emission wavelength of 700 nm with an IVIS spectrum. A shown in Fig. 2a and b, we identified quantitatively that Ce6-P_EGFRvIII_ signals by IN administration were detected stronger than those by IV or IP injection. After that, administration of Ce6-P_EGFRvIII_ and FlaB was proceeded by IN administration following the treatment schedule. Tumor formation was verified by MRI and H&E staining at 2 weeks after tumor cell implantation (Fig. 2c). Signals of Ce6-P_EGFRvIII_ in the brain were identified at the same region as the tumor site. FlaB was also confirmed to locate near the brain tumor by IF staining (Fig. 2c).

### P_EGFRvIII_ with FlaBenhances proportion of CD8 + T cells inthe brain and lymph node

**Fig. 3:**
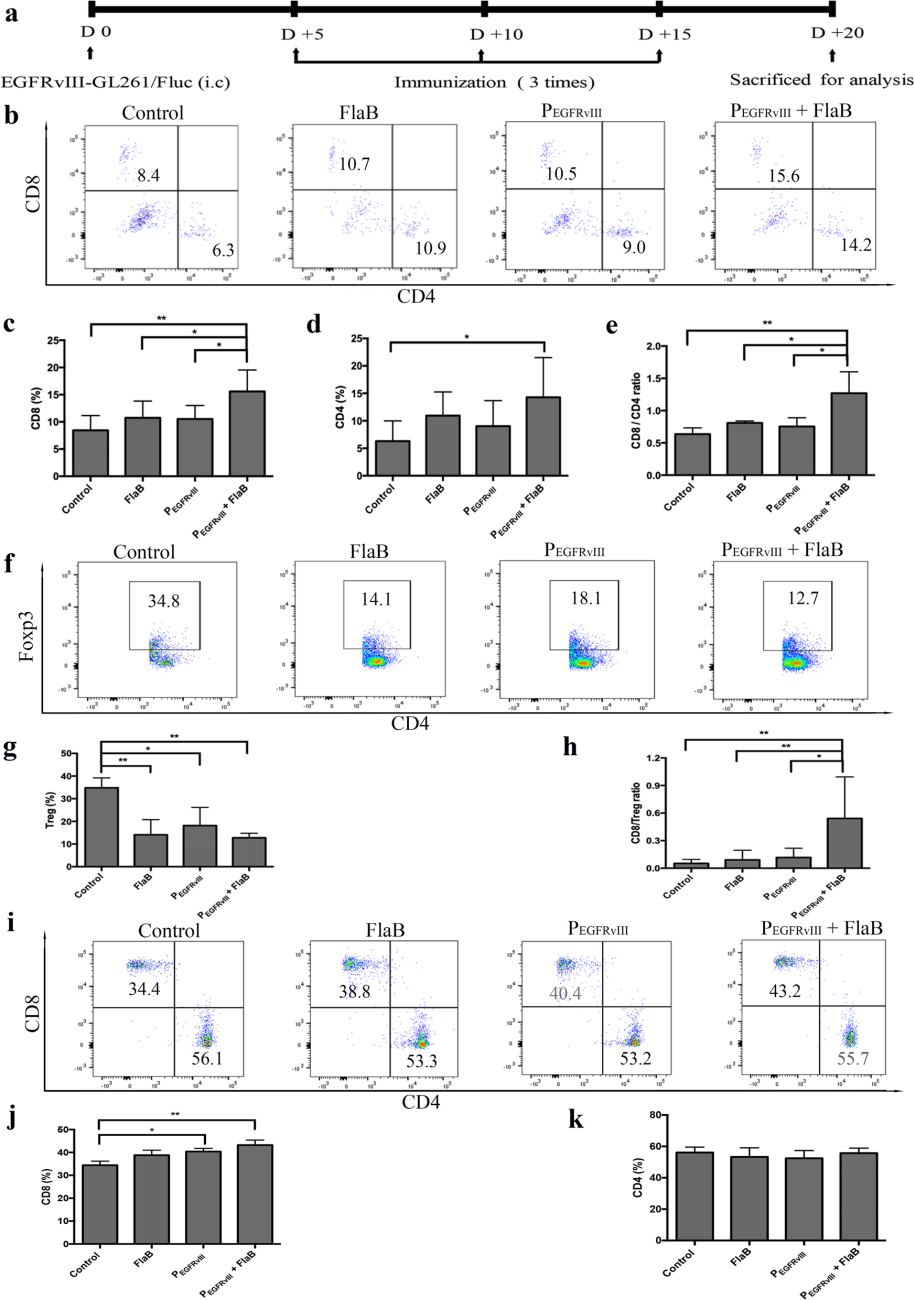
Distribution of T lymphocytes in brain tumor and cervical lymph node. **a** Mice were sacrificed on day 20 of the experimental treatment schedule. Mice were intranasally administrated with PBS, 5 ug FlaB alone, 15 ug P_EGFRvIII_ alone, or 5 ug FlaB plus 15 ug P_EGFRvIII_. Immunization was performed 3 times at intervals of 5 days. Single immune cells are isolated from mouse whole brain and cervical lymph node for flow cytometry analysis. **b**-**d** and **f**-**g** After gating CD3 + lymphocytes, the distribution of CD8 + and CD4 + T cells in brain tumors was analyzed. **e** and **h** Ratio of CD8/CD4 and CD8 / Treg cells in control and treated mice groups were estimated. **i**-**k** Distribution of CD8 + and CD4 + T cells in cervical lymph nodes was analyzed. Statistic results and graphs are presented as the mean of three independent experiments (*n* = 9 in each group). Significant differences are indicated by asterisk: *: *p* < 0.05, **: *p* < 0.01

FlaB mediates immune response through activation of TLR5-positive immune cells to a tumor in the host [13–15]. We investigated the population of T lymphocytes to identify the role of FlaB in immunotherapy using a GBM mouse brain tumor model. After cell implantation into the brain, immunizations were performed according to the treatment schedule (Fig. 3a). T cell population in the brain and lymph node was analyzed with isolated single cells by flow cytometry. In the brain, between control, FlaB alone, and P_EGFRvIII_ alone group showed no significant difference in the percentage of CD4 + or CD8 + cells. In contrast, the P_EGFRvIII_ plus FlaB group showed a significantly higher percentage of CD8 + cell population than the FlaB alone group (*p* = 0.0312) and the peptide alone group (*p* = 0.0235). CD8 + /CD4 + T cell ratio was also increased in the P_EGFRvIII_ plus FlaB group than in the FlaB alone group (*p* = 0.0223) and the P_EGFRvIII_ alone group (*p* = 0.0172) (Fig. 3b-e). And, the percentage of CD4 + Foxp3 + regulatory T cells (Treg) was decreased in the P_EGFRvIII_ plus FlaB group than in the control group (*p* = 0.068) (Fig. 3f and g). CD8 + /Treg ratio in the brain was significantly improved in the P_EGFRvIII_ plus FlaB group than in the FlaB and the P_EGFRvIII_ alone group (Fig. 3h). In the lymph node, the percentage of CD8 + T cells population was also significantly increased in the P_EGFRvIII_ plus FlaB group than in the control group (*p* = 0.0020). However, the proportion of CD4 + T cells showed no difference between the control and treated groups (Fig. 3i-k). These results indicate that P_EGFRvIII_ with FlaB could synergistically induce the population of CD8 + T cells in the brain and lymph nodes.

### FlaB enhances thetherapeutic efficacy ofP_EGFRvIII_ in a mousebrain tumor model

**Fig. 4:**
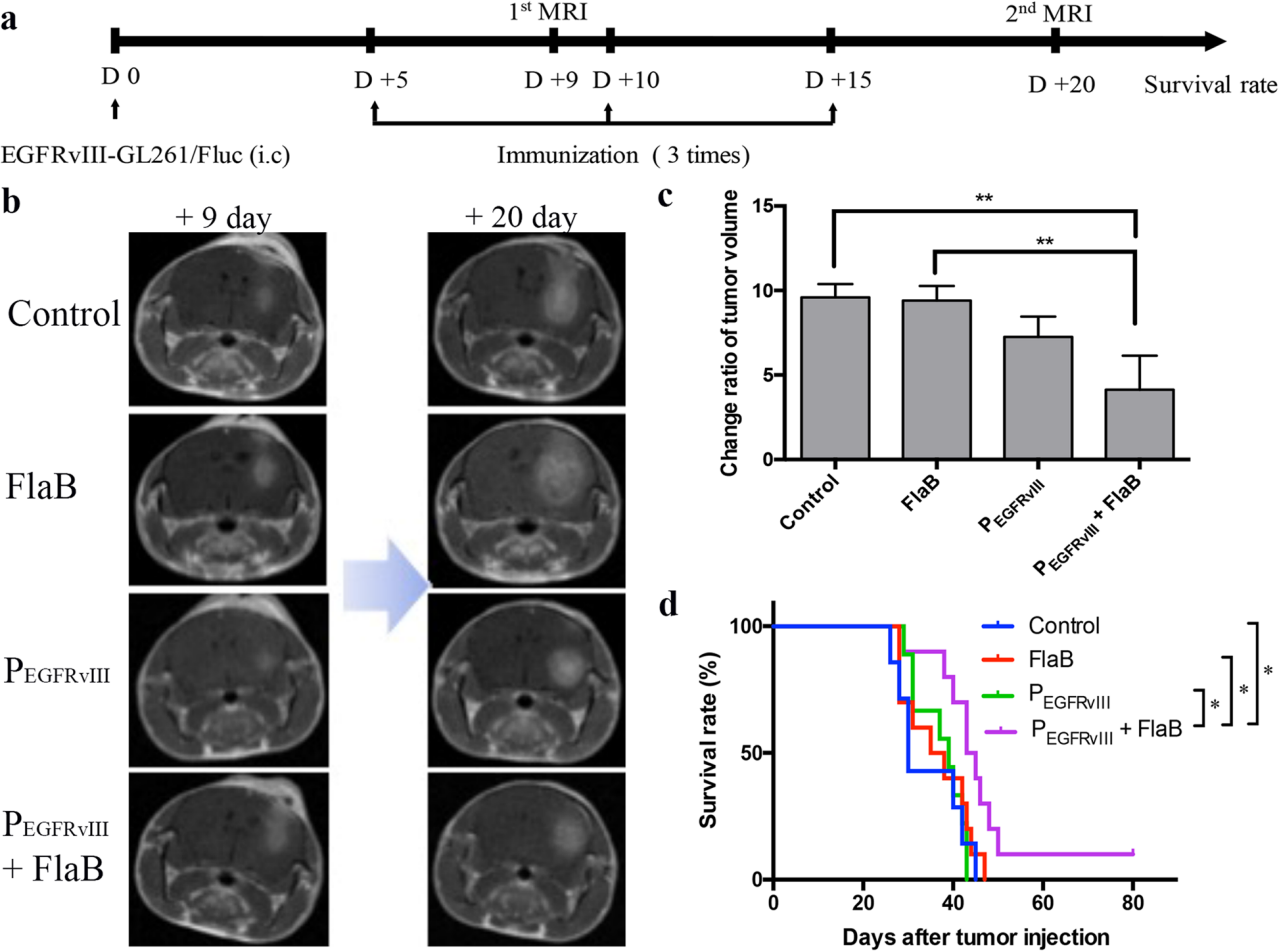
Tumor growth and survival rate based on an experimental treatment schedule. **a** Survival period of mice was proceeded until 80 days according to a study schedule. **b** T1 images of tumor at 9 days and 20 days by magnetic resonance imaging (MRI) are shown. The image represents one of three mice.** c** Tumor volume was measured by Osirix Lite v.12.0.1. (*n* = 3, **: *p* < 0.01). **d** Survival rates of mice in four groups: Control (*n* = 7), FlaB alone (*n* = 10), P_EGFRvIII_ alone (*n* = 9), P_EGFRvIII_ plus FlaB (*n* = 10). Results are presented as an average of three independent experiments. The survival curve was made by the Kaplan–Meier. Statistical significance was determined by a log-rank test (*: *p* < 0.05)

On day 9 and day 20 after cell injection based on the study schedule, MRI was performed to determine changes in tumor growth according to treatment (Fig. 4a). Figure 4b shows representative mouse brain MRI on control and treated groups. The tumor change ratio was measured to be 9.58 for the control group, 9.40 for the FlaB alone group, 7.25 for the P_EGFRvIII_ alone group_,_ and 4.13 for the P_EGFRvIII_ plus FlaB group (Fig. 4c). The FlaB alone group exhibited no tumor inhibition effect compared to the control group. The P_EGFRvIII_ alone group showed the inhibited tumor growth of about 25% than the control or the FlaB alone group. The P_EGFRvIII_ plus FlaB group showed inhibited tumor growth of about 65% compared to the control or FlaB alone group. With a change of tumor growth, mean survival was enhanced from 34.4 ± 2.9 days in the control group to 36.4 ± 2.3 days in the FlaB alone group, 37 ± 1.8 days in the P_EGFRvIII_ alone group (Fig. 4d), and 46 ± 4.0 days in the P_EGFRvIII_ plus FlaB group. These results revealed that P_EGFRvIII_ with FlaB could improve survival with a delay of tumor growth.

**Fig. 5:**
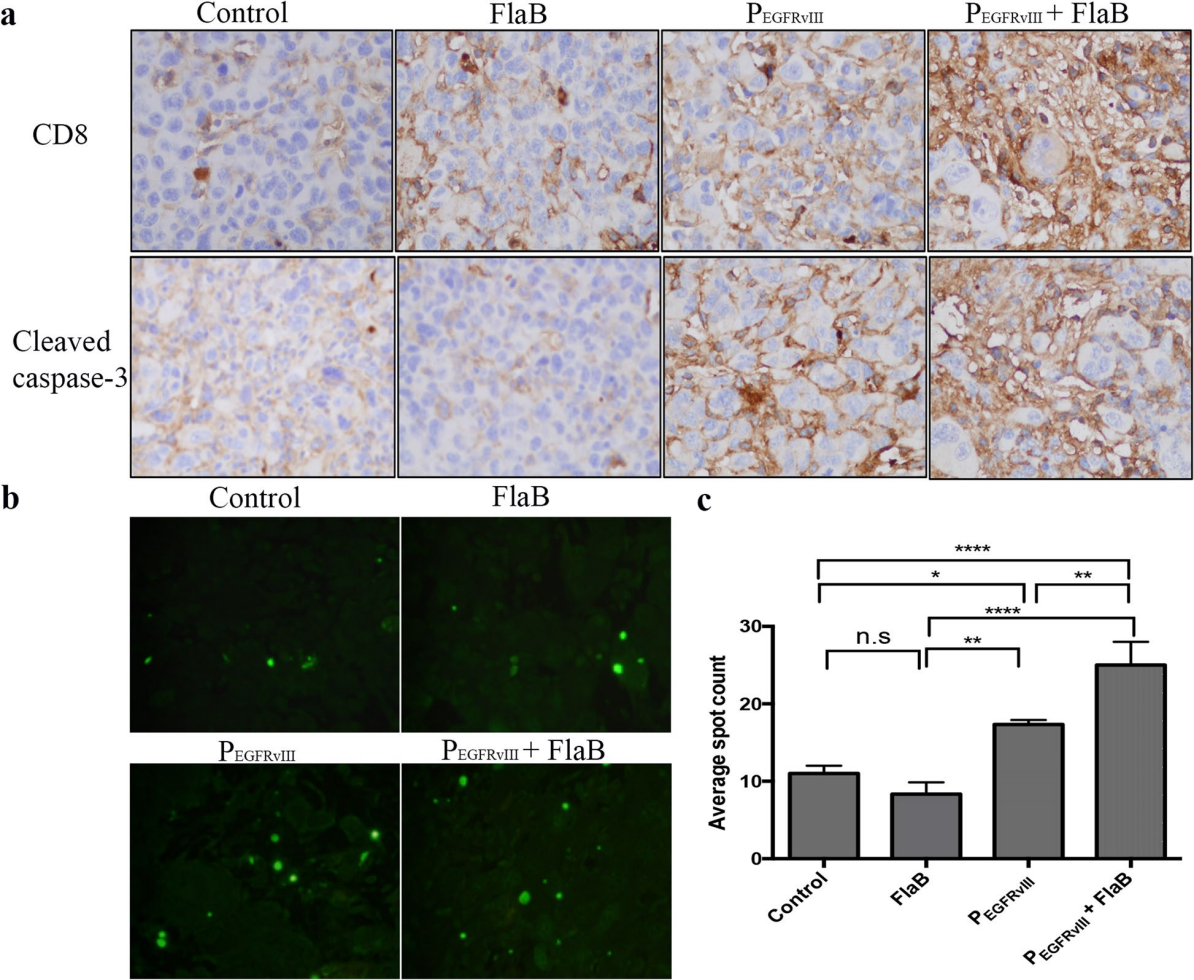
Induction of apoptosis in brain tumor tissues. **a** Mice were sacrificed on day 20 for paraffin-embedded tissue. Magnification: × 200. Distribution of CD8 + lymphocytes and expression of cleaved caspase-3 in extracted mouse brain tumors after IHC staining. Each immunostaining pattern was confirmed by triplicate specimens from the same mouse and typical image was shown in Fig. 5a. **b** DNA destruction in brain tumor tissues after TUNEL assay. **c** Spots were randomly selected from three regions in tumor tissues and counted to obtain an average fluorescence signal. Significant differences are indicated by asterisk (*: *p* < 0.05., **: *p* < 0.01., ****: *p* < 0.0001)

Infiltrated CD8 + T lymphocytes to tumors play a very important role in the anti-tumor effect for various cancers [23]. Thus, we investigated CD8 + lymphocytes and cleaved caspase-3 on tissue sections by IHC staining to determine the relation between CD8 + lymphocytes and tumor cell death for antitumor effect. Immunostaining levels of TIL CD8 + lymphocytes were increased in the P_EGFRvIII_ plus FlaB group compared to those in other groups (Fig. 5a). Expression levels of cleaved caspase-3 as an apoptosis marker were also consistently increased in the P_EGFRvIII_ plus FlaB group, like TIL CD8 + lymphocytes (Fig. 5a, supple Fig. 2b). In addition, DNA destruction showed the increased average spot number in the P_EGFRvIII_ plus FlaB group by using terminal deoxynucleotidyl transferase dUTP nick end labeling (TUNEL) assay (Fig. 5b and c). These results suggest the possibility that tumor cell death might be enhanced through increased infiltration of CD8 + lymphocytes in the P_EGFRvIII_ plus FlaB group.

## Discussion

GBM is one of the most difficult cancer for treatment due to its strong invasive and heterogeneous properties. Primary GBM is an isocitrate dehydrogenase (IDH)-wildtype and is associated with mutations of genes such as *EGFRvIII, TERT* promoter*, TP53,* and *PTEN* [24]. Among mutations, EGFRvIII-positive GBM has shown slight increases of PFS and OS by immunotherapy using EGFRvIII peptides in preclinical and clinical trials [10–12, 25, 26]. Although EGFRvIII-positive cells in GBM patients were removed by clinical trials (ACTIVATE, ACT II, ACT III), absolute treatment of GBM has a limitation due to recurrence by escape through the selection and proliferation of EGFRvIII-negative cells. Adjuvants are usually used to improve the therapeutic effect through immune response boosting of drugs including peptide vaccine. KLH conjugated EGFRvIII peptide (Rindopepimut) showed an increase in OS and PFS through potently immunogenic on GBM patients [26]. Among various adjuvants, flagellin is known as an adjuvant for immunotherapy that can induce increases of CD4 + and CD8 T + cells for various cancers including melanoma, colon, and breast cancers [19, 20, 27]. In our study, we hypothesized that FlaB could enhance peptide-based immunotherapy as an adjuvant or an immune-modulator in the glioblastoma-bearing mouse brain tumor model. We preferentially administered P_EGFRvIII_ and FlaB by IN as it was identified as the most effective delivery method. They were also located in brain tumor tissues. This indicates that P_EGFRvIII_ and FlaB administrated by IN can effectively provide an opportunity to interact with immune cells in the brain tumor environment. Although FlaB alone group showed no difference in the effect on tumor growth compared to the control group, peptide plus FlaB inhibited tumor growth more than P_EGFRvIII_ alone and FlaB alone. CD8 + lymphocytes and cleaved caspase-3 were histologically identified to have increased expression in the P_EGFRvIII_ plus FlaB group. This suggests that cleaved caspase-3 for tumor apoptosis and increase of CD8 + lymphocytes might be correlated.

GBM has a ‘cold’ tumor microenvironment that is formed through immune evasion by immune-suppressive factors such as gangliosides, kynurenine, TGF-β, IL-10, vascular endothelial growth factor (VEGF), and immunosuppressive cells such as Treg and MDSC. This tumor microenvironment can inhibit the CTL function of tumor-infiltrated T lymphocytes (TILs) and promote tumor growth by triggering T cell aging, tolerance, inability, and exhaustion [28–30]. Among TILs, reduction of CTL-trafficking to the tumor microenvironment and increase of Treg can occur by some factors [31, 32]. Inversely, an increase of CTL-trafficking into a tumor is associated with prolonged survival of GBM patients through tumor killing by CTL [33]. In various cancers, CTL is correlated with improvement of anti-tumor response [23, 34–36]. From this point of view, we assume that change of T cell population in the brain could influence tumor growth and survival of a GBM mouse model. In our study, the P_EGFRvIII_ plus FlaB group showed an increased proportion of CD8 + T cells in mouse brain tumors compared to other groups. However, the proportion of CD4 + FoxP3 + regulatory T cells was decreased in the P_EGFRvIII_ plus FlaB group compared to control or single treatment groups. This means that P_EGFRvIII_ plus FlaB can induce the brain tumor microenvironment to have CTL response condition by increasing CD8 + T cells and decreasing Treg. Flagellin can directly interact with CD11c + and CD3 + immune cells in both vagina and draining genital lymph nodes (gLNs) in a genital cancer mouse model [15]. Thus, an increase of CD8 + T lymphocytes in the brain might enhance cytotoxic T cells by modulating antigen-presenting immune cells in lymph nodes. In our study, peptide therapy with flagellin on GBM treatment was not enough for complete conquest due to its heterogeneity. To resolve this difficulty, combination therapy needs to as further study with radiation and checkpoint inhibitors.

## Conclusions

In summary, IN administration by inhalation can efficiently deliver FlaB and P_EGFRvIII_ to the brain. P_EGFRvIII_ with FlaB can induce an increase in CD8 + T cells and a decrease in Treg cells in a brain tumor. The induction of tumor apoptosis through the increase of CD8 + T cells can delay tumor growth and increase survival. Results of our study suggest that FlaB can serve as a potentiated adjuvant for the peptide-based vaccine in the GBM mouse brain tumor model.

## Supplementary Information

**Additional file 1: Supplementary figure 1.** Result of western blot. a. Exposure time was detected automatically (β-actin:6.4sec, AKT:5.1sec, pAKT: 1min 21.4sec, PI3K: 4.5sec, p-PI3K: 3 min). Primary antibody was used seperately after membrane cutting. b. Expressed proteins were quantificated by ImageJ 1.53e. (****: *p* < 0.0001, ***: *p* <0.001). **Supplementary figure 2.** Distribution of CD8+ lymphocytes and expression of cleaved caspase-3 in extracted mouse brain tumors after IHC staining. a. Each immunostaining pattern was confirmed by triplicate specimens from the same mouse and typical image was shown Fig.5a and supplementary Fig.2a. Magnification: x400. b. The quantitative analysis of CD8 and CC3 IHC score was shown. (****: *p* < 0.0001, **: *p* <0.005, *: *p*<0.05).

## Data Availability

The datasets generated and analysed during the current study are available from the corresponding author on reasonable request.

## Abbreviations

GBM: Glioblastoma
EGFRvIII: Epidermal growth factor receptor variant III
FlaB: Flagellin B
P_EGFRvIII_: EGFRvIII peptide
MRI: Magnetic resonance imaging
IHC: Immunohistochemistry
MDSC: Myeloid-derived suppressor cells
TGF-β: Transforming growth factor-β
DC: Dendritic cell
CAR: Chimeric antigen receptor
TLR5: Toll-like receptor 5
TCL: Tumor cell lysate
Ce6: Chlorin e6
NHS: N-hydroxysuccinimide
