# Supplementary material for: Flagellin synergistically enhances anti-tumor effect of EGFRvIII peptide in a glioblastoma-bearing mouse brain tumor model
Source: BMC Cancer. 2022 Sep 15;22:986. doi: 10.1186/s12885-022-10023-6 (PMC9479269; doi:10.1186/s12885-022-10023-6)
Supplement: Supplementary file 1 — Additional file 1: Supplementary figure 1. Result of western blot. a. Exposure time was detected automatically (β-actin:6.4sec, AKT:5.1sec, pAKT: 1min 21.4sec, PI3K: 4.5sec, p-PI3K: 3 min). Primary antibody was used seperately after membrane cutting. b. Expressed proteins were quantificated by ImageJ 1.53e. (****: p < 0.0001, ***: p <0.001). Supplementary figure 2. Distribution of CD8+ lymphocytes and expression of cleaved caspase-3 in extracted mouse brain tumors after IHC staining. a. Each immunostaining pattern was confirmed by triplicate specimens from the same mouse and typical image was shown Fig.5a and supplementary Fig.2a. Magnification: x400. b. The quantitative analysis of CD8 and CC3 IHC score was shown. (****: p < 0.0001, **: p <0.005, *: p<0.05). [file 12885_2022_10023_MOESM1_ESM.docx]

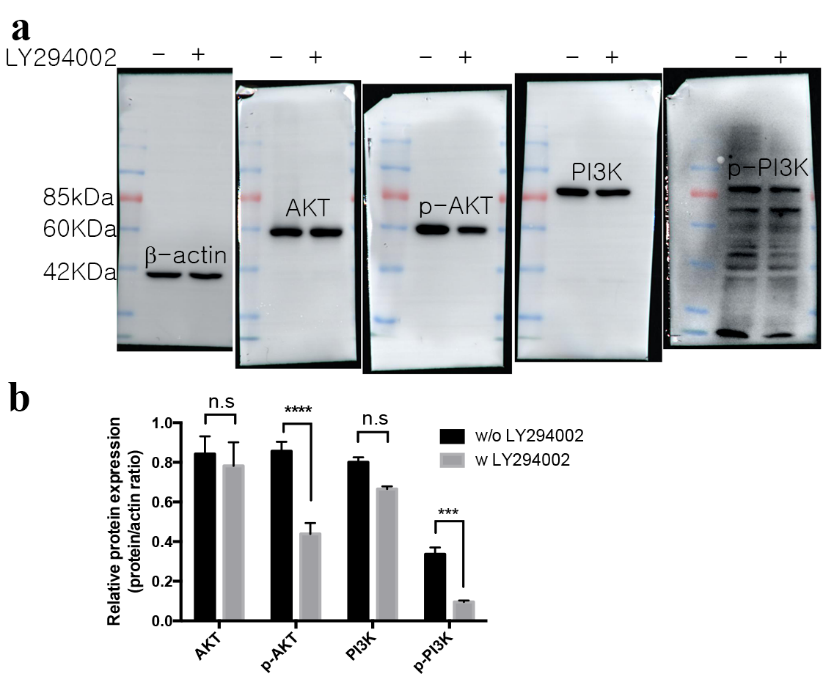


Supplementary figure 1. Result of western blot. a. Exposure time was detected automatically (β-actin:6.4sec, AKT:5.1sec, pAKT: 1min 21.4sec, PI3K: 4.5sec, p-PI3K: 3 min). Primary antibody was used seperately after membrane cutting. b. Expressed proteins were quantificated by ImageJ 1.53e. (****: *p* < 0.0001, ***: *p* <0.001)


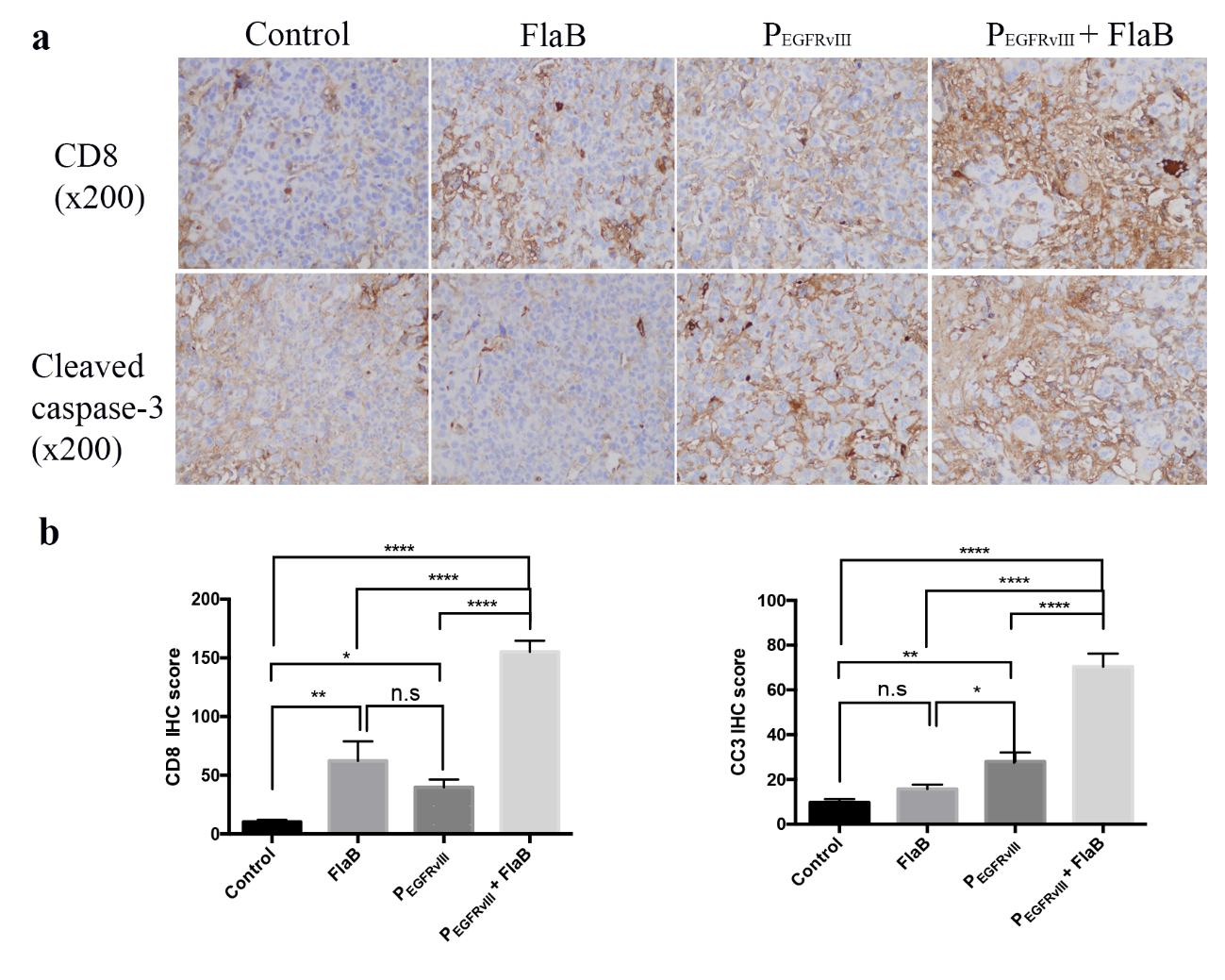


Supplementary figure 2. Distribution of CD8+ lymphocytes and expression of cleaved caspase-3 in extracted mouse brain tumors after IHC staining. a. Each immunostaining pattern was confirmed by triplicate specimens from the same mouse and typical image was shown Fig.5a and supplementary Fig.2a. . Magnification: x400. b. The quantitative analysis of CD8 and CC3 IHC score was shown. (****: *p* < 0.0001, **: *p* <0.005, *: p<0.05)
